# *Arcobacteraceae* comparative genome analysis demonstrates genome heterogeneity and reduction in species isolated from animals and associated with human illness

**DOI:** 10.1016/j.heliyon.2023.e17652

**Published:** 2023-06-27

**Authors:** Davide Buzzanca, Pieter-Jan Kerkhof, Valentina Alessandria, Kalliopi Rantsiou, Kurt Houf

**Affiliations:** a Department of Veterinary and Biosciences, Faculty of Veterinary Medicine, Ghent University, Heidestraat 19, Merelbeke, Belgium; b Department of Agricultural, Forest and Food Sciences (DISAFA), University of Turin, Largo Paolo Braccini 2, 10095 Grugliasco (TO), Italy; c Laboratory of Microbiology, Department of Biochemistry and Microbiology, Faculty of Sciences, Ghent University, Karel Lodewijk Ledeganckstraat 35, 9000 Ghent, Belgium

**Keywords:** *Arcobacter*, Gram negative, Genomics, Bacterial genomes, Virulence genes

## Abstract

The *Arcobacteraceae* family groups Gram-negative bacterial species previously included in the family *Campylobacteraceae*. These species of which some are considered foodborne pathogens, have been isolated from different environmental niches and hosts. They have been isolated from various types of foods, though predominantly from food of animal origin, as well as from stool of humans with enteritis. Their different abilities to survive in different hosts and environments suggest an evolutionary pressure with consequent variation in their genome content. Moreover, their different physiological and genomic characteristics led to the recent proposal to create new genera within this family, which is however criticized due to the lack of discriminatory features and biological and clinical relevance. Aims of the present study were to assess the *Arcobacteraceae* pangenome, and to characterize existing similarities and differences in 20 validly described species. For this, analysis has been conducted on the genomes of the corresponding type strains obtained by Illumina sequencing, applying several bioinformatic tools. Results of the present study do not support the proposed division into different genera and revealed the presence of pangenome partitions with numbers comparable to other Gram-negative bacteria genera, such as *Campylobacter*. Different gene class compositions in animal and human-associated species are present, including a higher percentage of virulence-related gene classes such as cell motility genes. The adaptation to environmental and/or host conditions of some species was identified by the presence of specific genes. Furthermore, a division into pathogenic and non-pathogenic species is suggested, which can support future research on food safety and public health.

## 1 Introduction

The recently proposed family *Arcobacteraceae* includes Gram-negative bacterial species isolated from different environmental niches and hosts [1,2]. The species included have been reclassified from the original genus *Arcobacter* of the family *Campylobacteraceae* due to their genomic differences observed in the past years. Their different sources of isolation and genomic heterogeneity has even led to the proposal of six genera and one candidate within the family *Arcobacteraceae* [1]. Although there is an increasing amount of information on the genomic features of some species belonging to the *Arcobacteraceae*, the genomic analysis is predominantly focused on the potential pathogenicity and general phylogeny, often leading to conflicting conclusions between authors [1,3]. In particular the division of the original genus *Arcobacter* into six genera: *Aliarcobacter, Halarcobacter, Malaciobacter, Pseudarcobacter, Poseidonibacter* and *Arcobacter* is still heavily discussed [1,3,4]. Recently, some authors have proposed to re-group these bacterial genera again into the original single genus “*Arcobacter*”, as the division does not reflect significant biological or clinical features. On et al. [3,4] came also to this conclusion after the analysis of average amino acid identity, average nucleotide identity, percentage of conserved proteins, alignment fractions, G-C percentages, *in silico* DNA–DNA hybridization values and genome-wide average nucleotide identity. For this reason, the term “*Arcobacter*” will be used in the present study to indicate the genus of the species belonging to the *Arcobacteraceae* family.

Some species are exclusively isolated from environmental matrices so far, such as from water sources, while others such as *Arcobacter bivalviorum, Arcobacter mytili*, *Arcobacter molluscorum* and *Arcobacter venerupis* have been collected from seafood such as mussels [2,[5], [6], [7]]. Some species, in particular those included in the newly proposed genus *Aliarcobacter,* are present in farm animals, in which they were initially associated with illness causing diarrhea, abortion and mastitis, although currently considered as non pathogenic for animals [2,8]. However, the species *Arcobacter butzleri, Arcobacter cryaerophilus, Arcobacter thereius*, and *Arcobacter skirrowii* have been isolated from diarrheic human stool samples and cases of septicemia. Food of animal origin and drinking water have been suggested as sources of infection [9,10]. The species *A. butzleri*, *Arcobacter cibarius*, *A. cryaerophilus*, *Arcobacter faecis, Arcobacter lanthieri, A. skirrowii, A. thereius*, and *Arcobacter trophiarum* seem to be commonly present in the intestinal track of livestock, causing no clinical symptoms nor reduced production parameters. After food processing, they may be present at levels up to 1000 colony forming units per gram on meat and meat products [2,11]. *A. cryaerophilus* and *A. butzleri* have also been isolated from other matrices, including raw milk and drinking water, and represent the species most commonly isolated so far [2,9,12,13].

At present, comparative genomics has been performed on only few *Arcobacteraceae* species, and current taxonomic debate supports the necessity of a broader evaluation of this bacterial family [[14], [15], [16]]. Therefore, the aim of the present study is a comparative genome analysis of the *Arcobacteraceae* family members, in particular on the clinically relevant species. More specifically the genomes analysis allows the detection of possible genome traits correlated to *Arcobacter* groups present in animals, which are considered as food-borne pathogens. This study asses the information obtained about *Arcobacter* pangenome to understand overall possible genomic relationships and the extent of genomic partitions (eg. core and accessory gene) among the *Arcobacter* groups proposed by Pérez-Cataluña and colleagues [1]. As only limited or even no additional isolates, other than the type strains, are available for the majority of the *Arcobacteraceae* species, the analysis were performed on type strains, as this approach allows more reliable assessment of the *Arcobacteraceae* pangenome because inclusion of multiple isolate genomes from only part of the species would lead to biased data on different pangenome partitions. This aspect was also considered for the selection of the species. The inclusion of only a part of the non-human and animals associated species has allowed the comparison between groups numerically comparable to mainly obtain information regarding the comparison between species isolated from land animals and humans with species from other sources.

## 2 Material and methods

### 2.1 DNA extraction and genome sequencing

**Fig. 1:**
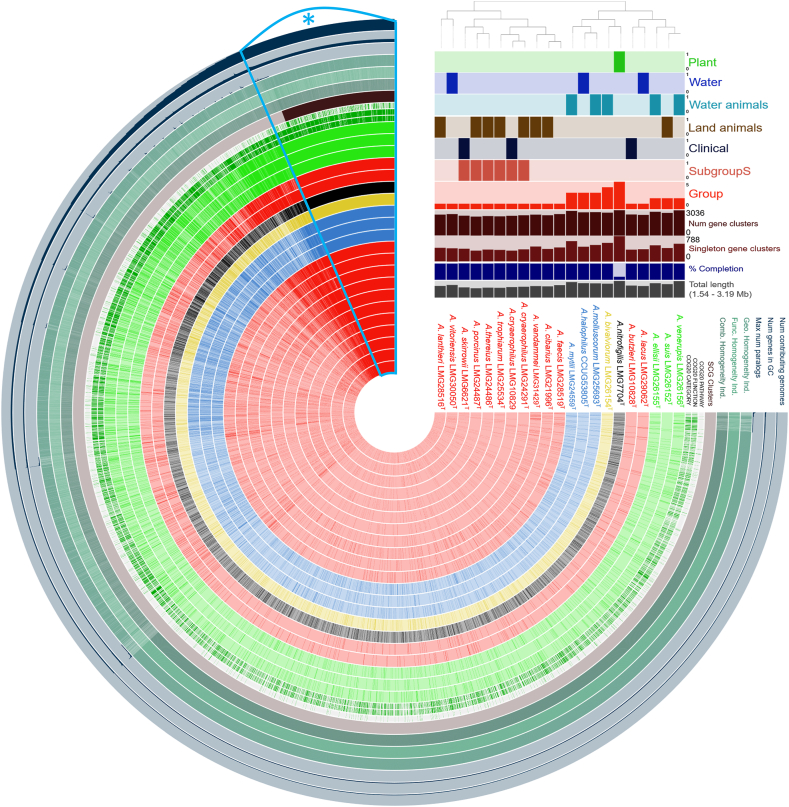
Analysis of gene clusters presence-absence in the *Arcobacter* species. The figure shows genomes characteristics of the *Arcobacter* species sorted for gene cluster presence and absence (species order). The strain isolation sources and the different species groups are displayed (presence/absence of bars; 1/0). Strains included in “clinical” were involved in clinical onset. The “num of contributing genomes” indicates the number of genomes where a gene cluster is present. The core genes are shown in the section indicated with the asterisk (*). The bars indicates difference information about the genomes. The bars above the names of the species, in addition to indicating the source of isolation, carry information regarding the genomes. The gray bars indicate the genome size (1.54–3.19 Mb), blue bars indicate completion percentages, dark brown bars indicate the number of gene clusters while light brown bars indicate the number of singleton gene clusters. (For interpretation of the references to color in this figure legend, the reader is referred to the Web version of this article.)

The species included as well as their characteristics, source of isolation, and their relevance in the clinical microbiology are shown in Fig. 1 with the *Arcobacteraceae* strains codes relating to the strains deposited at Laboratory of Microbiology, Department of Biochemistry and Microbiology, Faculty of Sciences of Ghent University bacteria collection (LMG; https://bccm.belspo.be/about-us/bccm-lmg) and Culture Collection University of Gothenburg (CCUG) (https://www.ccug.se/). The strains were stored in freeze-dried cultures (https://bccm.belspo.be/catalogues/lmg-catalogue-search). For genome sequencing, high-quality DNA extracts from 10^9^ bacterial cells in liquid cultures (Arcobacter broth; Oxoid; CM0965) were prepared using a Maxwell 16 tissue DNA purification kit (AS1030; Promega, Madison, WI, USA) and an automated Maxwell 16 DNA preparation instrument (AS2000; Promega). The final DNA extract was dissolved in 10 mM Tris-HCL pH 8.5 and was treated with RNAse (2 mg/ml, 5 μl per 100 μl extract). DNA quality was checked by 1% (wt/vol) agarose gel electrophoresis, and DNA purity was evaluated using the QuantiFluor One double-stranded DNA system and the Quantus fluorometer (Promega). Paired-end 2 × 150-bp libraries were prepared at the Wellcome Trust Human Genome Center (Oxford, UK) using a NEBNext DNA library kit for Illumina (New England Biolabs, Ipswich, MA, USA) and sequenced on an Illumina HiSeq 4000 instrument.

### 2.2 Genomes retrieval and assembly

Illumina paired-end accession numbers of the raw genome sequences are listed in Table 1. Part of these genomes have been obtained from the European nucleotide repository (https://www.ebi.ac.uk/ena/browser/home) as indicated in Table 1. Sequencing reads were prepared for assembly by adapter trimming and reads filtering using Trimmomatic v0.39 [17]. Reads with phred scores below 30 were removed, and nonpaired reads were discarded. The fastq files quality has been checked with the software fastqc v0.11.9 (https://www.bioinformatics.babraham.ac.uk/projects/fastqc/). The genomes were assembled with Shovill v1.0.4 (https://github.com/tseemann/shovill) a pipeline that includes selection of the best kmer and assembly based on SPAdes v3.15.2 [18]. After the assembly of the sequences, a quality check has been performed with Quast v5.0.2 [19]. The actual correspondence between genomes and different species has been evaluated using barrnap v0.9 with a similarity e-value cut-off of 1e-09 (https://github.com/tseemann/barrnap) to extract 16S rRNA gene sequences for a BLASTN 2.13.0+ comparison (https://blast.ncbi.nlm.nih.gov/Blast.cgi) and GTDB-Tk v1.7.0 for an entire genome evaluation [20]. BLASTN 2.13.0+ has been used for specific sequence comparisons. The Average Nucleotide Identity (ANI) has been calculated using OrthoANIu tool v1.2 [21].

**Table 1:** Information of the 20 *Arcobacteraceae* species included in the study, including the codes about Sequence Read Archive (SRA).

Complete species names	Strain	Group	Run accession
*Arcobacter butzleri*	LMG 10828^T^	1	SRR18076128
*Arcobacter cibarius*	LMG 21996^T^	1	SRR3664169*
*Arcobacter cryaerophilus*	LMG 24291T	1	SRR7985382*
*A. cryaerophilus*	LMG 10829	1	SRR7985571*
*Arcobacter porcinus*	LMG 24487^T^	1	SRR18076131
*Arcobacter skirrowii*	LMG 6621^T^	1	SRR18076130
*Arcobacter thereius*	LMG 24486^T^	1	SRR18076129
*Arcobacter trophiarum*	LMG 25534^T^	1	SRR18076127
*Arcobacter vandammei*	LMG 31429^T^	1	SRR18076126
*Arcobacter vitoriensis*	LMG 30050^T^	1	SRR18076123
*Arcobacter faecis*	LMG 28519^T^	1	SRR18076124
*Arcobacter lacus*	LMG 29062^T^	1	SRR5221256*
*Arcobacter lanthieri*	LMG 28516^T^	1	SRR18076125
*Arcobacter ellisii*	LMG 26155^T^	2	SRR7588928*
*Arcobacter venerupis*	LMG 26156^T^	2	SRR5914676*
*Arcobacter suis*	LMG 26152^T^	2	SRR7591528*
*Arcobacter halophilus*	CCUG 53805^T^	3	SRR7587110*
*Arcobacter molluscorum*	LMG 25693^T^	3	SRR7591199*
*Arcobacter mytili*	LMG 24559^T^	3	SRR7588217*
*Arcobacter bivalviorum*	LMG 26154^T^	4	SRR7586655*
*Arcobacter nitrofigilis*	LMG 7704^T^	5	NC_014166.1*
*Campylobacter jejuni*	NCTC 11168^T^	outgroup	NC_002163.1*
*Helicobacter pylori*	MT 5135^T^	outgroup	NZ_CP071982.1*

The accession numbers indicated with “*” have been retrieved from ENA (https://www.ebi.ac.uk/ena/browser/home). The strains sequenced in this work are available at the NCBI bioproject PRJNA808439. The column “group” reports the group of strains as indicated by Pérez-Cataluña et al. [1]. The genomes of *Campylobacter jejuni* and *Helicobacter pylori* type strains were included as outgroup [35].

### 2.3 Functional annotation and pathway annotation

The genomes have been annotated with the software Prokka v1.14.5 (database HAMAP, CMs: Bacteria, Kingdoms: Bacteria) to obtain sequences and functions of the genes (functional annotation), and to obtain input gene sequence files (e.g.gff, .fna, .faa) for subsequent bioinformatic analysis [22]. The pipeline KAAS v2.1 (https://www.genome.jp/kegg/kaas/) [23], MicrobeAnnotator v2.0.5 (considering completeness of pathway at least 80% in one genome, kofamscan and swissprot database) [24] and emapper-2.1.6 (http://eggnog-mapper.embl.de/) have been applied to obtain data about gene pathways of aminoacidic and nucleotide sequences [25]. CRISPR-Cas++ v1.1.2 has been employed to detect CAS and CRISPR sequences [26]. The detection of pathways linked to secondary metabolites production has been performed with antiSMASH 6.0.1 [27].

### 2.4 Pangenome evaluation tools

To assess the pangenome characteristics, the pangenome analysis tools Roary v3.13.0 [28] and Panaroo v1.2.8 (threshold ≥80% identity) have been applied [29]. They have been used on.gff files from Prokka annotation to identify the core and accessory gene partitions jointly at the obtainment of a binary matrix relating to gene presence/absence in the species. The bioinformatics tool PPanGGOLiN v1.1.136 has been applied with default options to identify additional data about pangenome partitions and genomic plasticity regions [30,31]. Graphical visualization of pangenomes partitions has been performed with Anvi'o v7.1 (mcl 8) [[32], [33], [34]]. These analyses have been performed on the 21 genomes, excluding the two outgroup species (*Campylobacter jejuni* and *Helicobacter pylori*) [15,35]. The orthogroups analysis has been performed with OrthoFinder v2.5.4 [36], whereas the possible correlation between the presence and absence of particular orthogroups within different strains and characteristics has been evaluated with Scoary v1.6.16 [37].

MegaX v10.1.7 [38] has been used to obtain dendrograms from specific sequences, while bcgTree v1.1.0 [39] has been applied to construct a phylogenetic tree based on 107 single-copy genes from core amino-acid sequences.

### 2.5 Statistical analysis and visualization

Homogeneity tests have been evaluated with Shapiro-Wilk's W and Modified Levene's tests (Brown-Forsythe test), while Kruskal–Wallis (K–W) and Anova test have been used to evaluate overall differences and variations between multiple groups. Wilcoxon Rank sum test (WRS) and two-sample *t*-test have been performed to evaluate differences between two groups, for nonparametric (K–W, WRS) and parametric data (Anova, *t*-test). A *post hoc* analyses Dunn's and Tukey's tests have been performed for nonparametric and parametric data respectively. All statistical analyses have been performed with RStudio 2021.09.0 (R v3.6.1, https://www.r-project.org/). The Spearman's correlation test has been performed with Past4 v4.09 [40]. The dendrogram trees have been constructed, visualized, and graphically curated with iTol online software (https://itol.embl.de/) [41].

### 2.6 Availability of data

The raw sequence reads of the genomes sequenced in the present study have been deposited on NCBI (https://www.ncbi.nlm.nih.gov/) at bioproject number PRJNA808439. The sequence codes obtained from the ENA database (https://www.ebi.ac.uk/ena/browser/home) are indicated in Table 1 together with the run raw sequence reads codes relating to the bioproject PRJNA808439. The genome assemblies are available on Zenodo (https://zenodo.org/; https://doi.org/10.5281/zenodo.6669654).

## 3 Results and discussion

### 3.1 *Arcobacteraceae* genomes analyses suggest the presence of a single genus

**Fig. 2:**
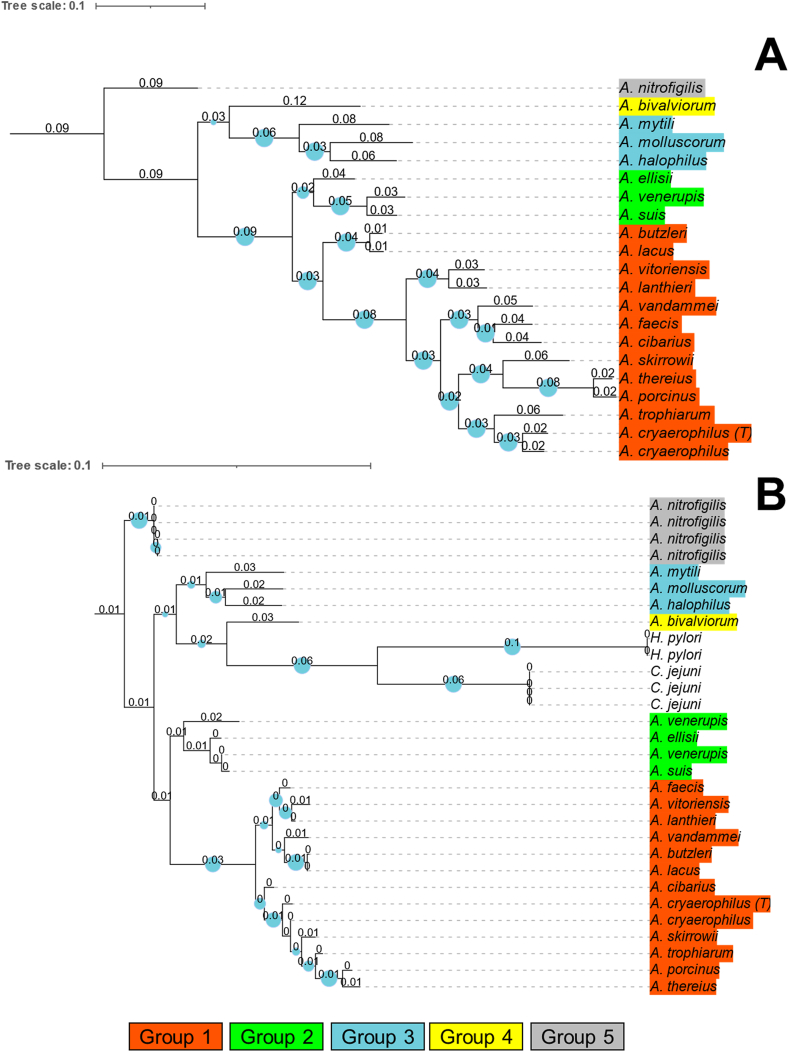
Dendrograms of *Arcobacter* species. The trees have been computed from different input sequences. The dendrogram in panel A was produced on amino acidic sequences from Prokka analyzed with bcgTree (107 core sequences). The scale bar and numbers represent the number of amino acid substitutions per site. The dendrogram B represents the maximum likelihood estimation obtained on 16S rRNA sequences (10.000 bootstraps). The species have been marked with different colors according to the group. (For interpretation of the references to color in this figure legend, the reader is referred to the Web version of this article.)

Information regarding the 20 *Arcobacter* type strains, as well as on the extra *A. cryaerophilus* strain, included to cover the heterogeneity of this species [42], are shown in Table 1 and Fig. 1. These type strains represent groups 1 to 5 as proposed by Pérez-Cataluña et al. [1]. The species *Arcobacter vandammei*, which was not yet described at that time, was placed in group 1, considering its characteristics and phylogenetic position (Fig. 2A and B) [43]. Though several species have been isolated from multiple sources and various hosts, the initial isolation source recorded for each type strain were considered. *A. cryaerophilus, A. lanthieri* and *A. thereius* that have been associated with human clinical cases were included in the “clinical” group in the genomic traits analysis, even if the reference strain was initially isolated from a non-human matrix (Fig. 1).

All genomes originate from the assembly of Illumina raw sequence reads (Supplementary Table 1). This strategy was followed in order to reduce differences related to the level of genome completeness and error rates deriving from the sequencing technologies [44,45]. Moreover, the use of raw reads provided assemblies from the same assembler [46]. The genome “normalization” (raw sequence reads from Illumina, use of the same assembler) has therefore made the comparison of bacteria possible with reduced technical differences. Nevertheless, the use of draft genomes does not provide all information that can be derived from complete genomes. However, the objectives of this study were linked to the detection of differences between groups considered phylogenetically different, and in this frame the use of draft genome has been commonly applied and accepted in previous studies [1,[47], [48], [49]]. For example, draft genomes have previously been used to detect strain level differences within some *Arcobacter* species [[14], [15], [16],^50^].

Genome comparison displays a significantly smaller genome size of the 12 species included in group 1 (2.12 Mb, st. dev ±0.2 Mb, *p-value* < *0.05*) compared to the species of the other four groups, though without a significant difference in the GC percentages. Moreover, the genomes of the species in group 1 show less genes than those in other 4 groups (average 2169 genes, st. dev ±156, *p-value* < *0.05*) (Supplementary Table 1). Group 1 includes strains isolated from mammals, of which some associated with human infections. In particular, the species *A. butzleri*, *A. cryaerophilus*, *A. lanthieri, A. skirrowii,* and *A. thereius* are currently considered emerging foodborne pathogens [9,51]. A smaller genome and a loss of genes is a phenomenon that has previously been reported in other pathogenic bacteria when compared to their nonpathogenic or less pathogenic relatives [52,53]. Furthermore, a subgroup of group 1 (henceforth called subgroup S, including *A. cryaerophilus*, *Arcobacter porcinus*, *A. skirrowii, A. thereius* and *A. trophiarum*), shows a reduced size of the genome and a smaller number of genes compared to the other seven species within group 1 (*p-value* < *0.05*) (Supplementary Table 1).

The gene class pathway analysis (KAAS, KEGG codes) of the orthogroups (OGs) revealed sequences belonging to 115 gene classes, of which the most abundant are linked to metabolic pathways (319 OGs), biosynthesis of amino acids (86 OGs), biosynthesis of cofactors (79 OGs), microbial metabolism in diverse environments (71 OGs), ribosome (51 OGs), and carbon metabolism (41 OGs). In agreement with these results, gene enrichment analysis, performed to obtain Clusters of Orthologous Genes (COGs) category codes, revealed the presence of orthogroups present in all 20 species related to translation, ribosomal structure, and biogenesis, amino acid transport and metabolism, coenzyme transport and metabolism, energy production and conversion (Supplementary Fig. 1). This suggests the presence of a core genome composed of genes with functions linked to fundamental metabolic functions. This aspect is predictable, considering the importance of these genes in different bacteria for their primary metabolism. Moreover, the importance of amino acid transport and metabolism as opposed to carbohydrate transport and metabolism (33 orthogroups, COGs) previously reported, has been confirmed by the present data, where many sequences related to amino acid metabolism were observed [9,16,54]. Importantly, the analysis showed a lack of information in the COGs database about specific orthogroups that result to be unknown (Supplementary Fig. 1). Further studies are required to elucidate the function of these genes.

Dendrograms constructed on amino acidic (107 core sequences; Figs. 2A) and 16S rRNA nucleotide sequences (1 sequence each genome; 4 sequences in *Arcobacter nitrofigilis*; similarity e-value cut-off of 1e-09; Fig. 2B) displayed the absence of a clear separation between the members of the 5 proposed groups. This observation is also supported by the dendrogram's different distances between and within the groups: in some cases, the genomic distance was higher within the same group than between different groups (Fig. 2A). Furthermore, the orthogroups and gene cluster analysis showed a separation of the species *A. butzleri* and *Arcobacter lacus* from their group (Fig. 1, Fig. 2A). These aspects indicate an absence of phylogenetic stability of the newly proposed genera, as also suggested by other authors [3,4].

The ANI is useful to obtain taxonomic information in prokaryotic genomes [55]. Barco et al. [56] observed an estimated genus boundary ANI mean points of 73.98% (median of 73.11%) from the analysis of more than 140 bacterial genera. The ANI analysis showed that *A. nitrofigilis* and *A. trophiarum* have the lowest percentage of similarity (73.23%) among the *Arcobacteraceae* genomes (Supplementary Fig. 2). *A. nitrofigilis* showed ANI percentages below 74% from the comparison with *A. cryaerophilus, A. porcinus, A. skirrowii, A. thereius* and *A. vandammei*, while *A. trophiarum* shows values below this percentage from the comparison with *A. bivalviorum, Arcobater halophilus* and *A. molluscorum*. The ANI values obtained from *Arcobacteraceae* species result above the threshold normally observed by Barco et al. [56], disagreed with the proposed groups regarding the subdivision of *Arcobacteraceae* family into multiple genera. The two *A. cryaerophilus* genomes showed an ANI value of 92.72% confirming the heterogeneity of this species [42].

### 3.2 Pangenomes partition shows a wide presence of persistent genes

**Fig. 3:**
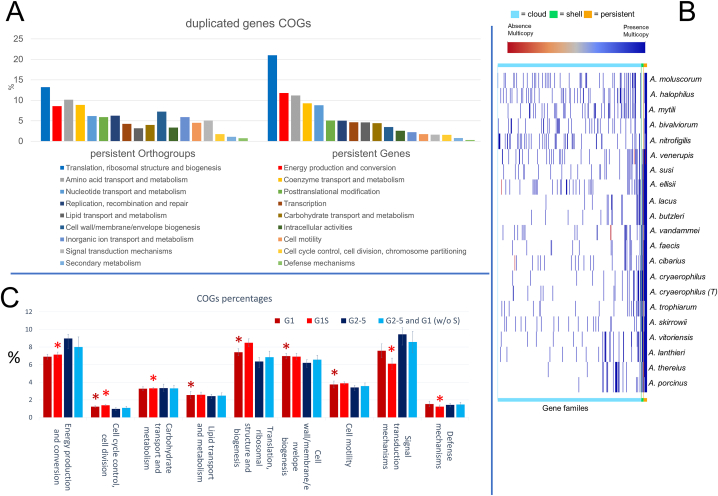
Pangenome analysis of the *Arcobacter* species. The histogram (A) shows the percentages of the different persistent OGs and gene pathway families. The panel B shows the presence of multiple genes copies (blue = present, red = absent). Most of the multiple copies are in the right part of the graph in correspondence of the genomic portion related to core and shell genome. The bar chart (C) shows the percentage of COGs classes with standard deviations. The groups shown are: group 1 (G1), group S (G1S), group 2–5 together (G2-5) and all groups excluding the subgroup S (G2-5 and G1 w/o S). The asterisks (*) indicates differences statistically significant (*p-value* < *0.05*) between G1 compared to G2-5 (dark red asterisks) and G1S compared to G2-5 and G1 w/o S (red asterisks). (For interpretation of the references to color in this figure legend, the reader is referred to the Web version of this article.)

Pangenome analysis of the 20 *Arcobacter* species has been performed on the gene sequences obtained during the functional annotation process. The result revealed the presence of 505 core genes with the tool Panaroo (Supplementary Fig. 3A). However, pangenome analysis performed with the tools Roary and PPanGGOLiN show only the presence of 296 and 269 core genes, respectively, demonstrating again the largely different outcomes obtained when different analysis tools are applied, as also previously reported [29,57] (Supplementary Fig. 3A). To evaluate these conflicting results, OGs (orthoFinder) and gene family comparison have been performed with orthoFinder and PPanGGOLiN, respectively. Results showed 1324 persistent (= present in a range between 90 and 99% of the species) OGs (32.19% of assigned OGs), 501 persistent gene families (average of the number of persistent families present in the 21 genomes) and a relevant number of gene families in cloud partition (average 1669 gene families) [31] (Supplementary Fig. 3B). The high number of cloud gene families, representing genes shared only between few genomes, demonstrates a flexible genome linked to the adaptation of the species at different environmental matrices and hosts. The analysis of the number of gene families among the strains led to observe a lower presence of persistent gene families in *A. nitrofigilis* (376) compared to the other species. This result agrees with the lower “completion” (based on presence or absence of Single-copy Core Genes; SCGs) of its genome from the analysis with Anvi'o software (Fig. 1) and confirmed by PPanGGOLiN completeness analysis (= 66%). The analysis of gene family duplication showed the presence of a wide number of genes in the persistent partition, leading to the hypothesis of a gene duplication strategy linked to a phenomenon of redundancy to keep certain metabolic functions active even in the event of mutations (Fig. 3A and B). This is linked to the gene enrichment results on persistent genes and GOs which detected a wide presence of sequences related to fundamental functions, most of which linked to translation, ribosomal structure and biogenesis, energy production and conversion and amino acid transport and metabolism (Fig. 3A). The GOs results support again the importance of amino acid metabolism for the metabolism of this bacterial family [9,16,54].

Results of the pangenome analysis are similar to what is reported in other pangenome studies, such as the pangenome partitions of *Campylobacter* or *Enterococcus* spp. [[58], [59], [60], [61]]. The core genes detected in *C. jejuni* and *Campylobacter coli* were 531, a number comparable to the core genes of *Arcobacter* considering the greater number of species analyzed [60]. The *Arcobacter* species pangenome analysis supports the presence of a single genus within the family, as also argued by On et al. [3].

### 3.3 Animal related species show a different gene classes composition

The significantly reduced genome size of the animal associated species in group 1 indicates the loss of genes and a link to their potential pathogenic nature. This aspect has also previously been reported for other pathogenic bacterial species [52,53]. The reduction of genome size is linked to an evolutionary adaptation to the host. Particularly relevant is the possibility to lose genes encoding proteins detected by the host immune system as well as the loss of specific pathways related to environmental survival and spreading [53,62]. To understand the relation between the absence of genes and certain characteristics, a comparison of the gene classes present and absent between the species with and without reduced genomes has been performed (Supplementary Table 2). Data of group 1 showed the presence of a smaller number of sequences related to amino acid transport and metabolism, energy production and conversion, carbohydrate transport and metabolism, intracellular trafficking, secretion and vesicular transport, post-translational modification, signal transduction mechanisms together with lipid transport and metabolism, defense mechanisms, cell motility, protein turnover, chaperones, cell wall-membrane-envelope biogenesis and translation, ribosomal structure and biogenesis (*p-value* < *0.05*). Part of these gene classes have also been lost in other pathogenic bacteria [53]. The lipid transport metabolism genes loss, together with the loss of cell wall and membrane envelope biogenesis genes, can be linked to an escape strategy from the host immune system changing part of the lipopolysaccharide (LPS) O-antigen structures. The loss of LPS-related genes has also been observed in pathogenic *Yersinia pestis* [53,63]. Genes linked to chemotaxis, energy production, carbohydrate, amino acid metabolism and flagella production can be lost during bacterial evolution to adapt to changing environmental niches and to host and host related environmental conditions [53].

However, the ratio of the orthogroups gene classes (= % of specific classes on COGs total) including lipid transport and metabolism, cell motility, cell wall-membrane-envelope biogenesis, translation, cell division, chromosome partitioning and ribosomal structure, biogenesis, and cell cycle control results higher in group 1 compared to the species of the groups 2 to 5 (*p-value* < *0.05*) (Fig. 3C). This suggests not a simple gene loss but an evolutionary adaptation to environmental and host conditions. The higher percentage of genes linked to lipid transport, metabolism and cell motility suggests a possible role during host colonization as previously reported for *A. butzleri* virulence capacity [14,16].

Five of the twelve species of group 1 (*A. cryaerophilus*, *A. porcinus*, *A. skirrowii, A. thereius* and *A. trophiarum*) have a reduced genome. Compared to the other seven species in group 1, these species (referred to as subgroup S) show a lower number of genes linked to energy production and conversion, carbohydrate transport and metabolism, defense mechanism, cell wall-membrane-envelope biogenesis and signal transduction mechanisms (*p-value* < *0.05*) (Fig. 3C). This suggests a further genome evolution adaptation. Moreover, the loss of these genes suggests the possibility of an importance underestimation of these species in veterinary and human clinical cases due to a reduced capability to grow on laboratory media conditions [64]. The comparison between species of the subgroup S and *A. butzleri,* shows a lack of group-specific carbohydrates-related genes in the subgroup S. Five OGs have been detected only in *A. butzleri*, these OGs are linked to ADP-glyceromanno-heptose-6-epimerase-activity, polysaccharide-deacetylase, haloacid-dehalogenase-like-hydrolase, membrane protein EamA and an ABC-transporter. Moreover, a carbohydrate-related major facilitator superfamily OG (Membrane transport protein) is present in subgroup S but absent in *A. butzleri*. The absence of these metabolic genes could explain the more difficult isolation of species belonging to subgroup S and consequently their underestimation in clinical cases.

### 3.4 Specific pathways linked to different *Arcobacteraceae* groups

**Fig. 4:**
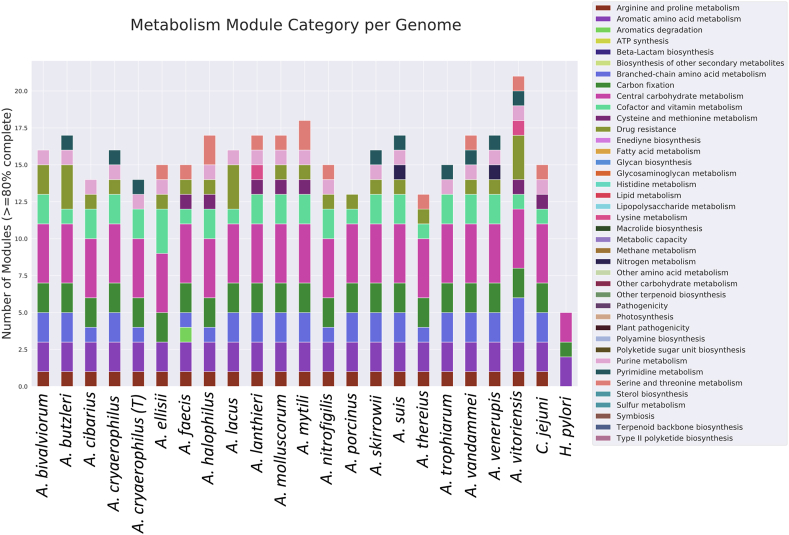
Genomes Annotated Pathway. The histogram shows the pathway modules with at least 80% of completeness in at least one genome (MicrobeAnnotator).

Species within the family *Arcobacteraceae* showed to have specific pathways correlated to different environmental conditions linked to isolation sources (Spearman's correlation of pathway completeness percentages, S. corr., *p-value* < *0.05*) (Fig. 4). Species within group 1 show a positive correlation (S. corr. 0.64) to carbapenem resistance demonstrating the presence of antibiotics resistance-related genes in the species often isolated from humans and mammals. The cobalamin biosynthesis and assimilatory nitrate reduction are negatively correlated to the group 1 (S. corr. −0.77, −0.71). This negative correlation was also observed to the subgroup S (S. corr. −0.46, −0.62). The different correlation among groups of cobalamin-related genes, suggests the opportunity of animal-related species to consume cobalamin from the host, as also reported for other pathogens [65]. Furthermore, cobalamin biosynthesis and assimilatory nitrate reduction pathway show a positive correlation in group 2 (S. corr. 0.62, 0.61 respectively), which includes species isolated from shellfish (*Arcobacter ellisi*, *A. venerupis*) and pork meat (*A. suis*), but not related to pathogenicity in humans and present in mammals, while lysine biosynthesis turns out negatively correlated (S. corr. −0.49). [63]. The species included in group 3, isolated from water environment and water animals, show a positive correlation with threonine and ectoine biosynthesis (S. corr. 0.58, 0.63) (Supplementary Table 3). Ectoine is an osmosis-stress protective molecule, and the presence of this pathway is not surprising considering their link to an environment characterized by a high salt concentration [66]. Interestingly, the ectoine pathway is negative correlated with the *Arcobacter* species of group 1 (S. corr. −0.49). Group 1 (containing species related to animals and humans) shows the presence of thiopeptide-related genes. These genes are linked to the production of thiopeptide, an antibiotic active against Gram-positive bacteria [67]. The presence of these sequences has been observed in nine of the twelve species of group 1.

Another metabolic pathway linked to a particular species ecological niche is related to phthoxazolin (NRPS-T1PKS) [68,69]. This metabolite is a cellulose inhibitor, and its presence in *A. nitrofigilis,* bacteria associated with *Spartina alterniflora,* suggests a role in bacteria-plant interaction [70].

Due to the high presence of hypothetical proteins, it is complicated to evaluate the presence of complete pathways. However, the results so far show specialization to a certain extend of some *Arcobacter* species with different environmental and host characteristics.

### 3.5 Putative virulence genes are not strongly correlated to different groups

**Fig. 5:**
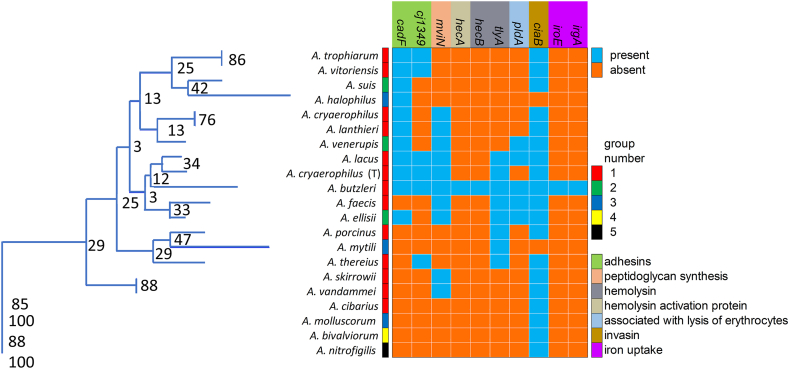
PVGs presence-absence dendrogram. The figure shows the dendrogram produced on the presence-absence binary matrix of ten genes currently considered virulence-related. The tree (Jaccard, Neighbor-joining) shows bootstrap value (10.000) while the different colors near species names indicate the species belonging group. (For interpretation of the references to color in this figure legend, the reader is referred to the Web version of this article.)

In most of the *Arcobacter* pathogenicity studies, the presence of 10 putative virulence genes (PVGs), (*iroE, hecA, hecB, ciaB, cadF, cj1349, irgA, pldA, mviN* and *tlyA*) is assessed [[71], [72], [73], [74], [75]]. As already stated above, some *Arcobacter* species are linked to infection in humans and are present in mammals, most of these species are included in group 1. The 20 species included in the present study harbored at least one of these virulence associated genes (Fig. 5). The comparison of the presence of PVGs in the different groups (comparison with LMG 10828^T^, BLAST query coverage ≥80% and percent of identity ≥95%) shows that few traits are associated to specific groups (Spearman's correlation *p-value* < *0.05*). The comparison was performed between genes from LMG 10828^T^ and the others *Arcobacteraceae* genomes. *A. butzleri* was chosen for comparison as the most studied pathogen of this family with most information on these PVGs [16,71,72]. The data shown in Fig. 5 are related to the presence/absence of gene variants comparable to *A. butzleri* PVGs and conserved in the *Arcobacteraceae* family not excluding the presence of others genetic variants. The variability of these sequences was also confirmed by genome analysis on *A. butzleri* strains [16,75] and on genomes of other species [[73], [74], [75]]. An example is the genes *tlyA* of *A. faecis* LMG 28519^T^ the presence/absence of which has led to conflicting results among authors [73,74]. The PVGs *cj1349* (fibronectin-binding protein) and *ciaB* (invasin) are positively correlated to group 1 and to clinical-related species (*A. butzleri, A. cryaerophilus, A. skirrowii, A. thereius, A. lanthieri*), respectively (0.49, 0.51 respectively). The gene *mviN* (virulence factor) is negatively correlated to group 3 that includes species isolated from marine animals and aquatic environments (−0.56). However, the absence of a strong association between PVGs and specific groups suggest the necessity to consider new genes in the study of *Arcobacteraceae* when focused on their virulence mechanism. No correlation was detected for gene clusters and orthogroups related to flagella that are considered involved in virulence functions (*p-value* > *0.05*). [15].

In the present study, different Clustered Regularly Interspaced Short Palindromic Repeats (CRISPRs) sequences have been detected. However, *A. butzleri*, *A. lacus* and *A. trophiarum* do not contain CRISPR and CAS sequences while the other species contain at least one (Supplementary Table 1). A degeneration of CRISPR-CAS sequences has been linked to an increased virulence and antibiotic resistance in different bacteria, including *C. jejuni* [76]. A lack of CRISPR–CAS sequences has been observed in the predominant species considered pathogenic, namely *A. butzleri,* together with a species with a high genome similarity, *A. lacus*. Although information about the pathogenicity of *A. lacus* are lacking. Moreover, *A. trophiarum*, which displayed an *in vitro* colonization and invasion behavior similar to *A. butzleri*, does not show a presence of CRISPR-CAS sequences [77].

The Scoary comparison between the five *Arcobacteraceae* groups*,* and between strains characterized by different sources of isolation, allowed only the detection of 47 group-correlated OGs in group 1 (Scoary, Bonferroni's and Benjamini-Hochberg's methods corrected, *p-value* < *0.05*), of which 8 orthogroups are positively correlated to group 1. Among these OGs, Hemolysins-CBS-domain genes orthogroup is present in all species belonging to group 1. As some of those species are considered pathogenic, the presence of this gene hypothesizes the role of hemolysis in their pathogenicity. The other genes related to group 1 are linked to different pathways, in particular to amino acid transport and metabolism (3 OGs positively correlated and 3 negatively correlated), while energy production and conversion is the class with the largest negatively correlated OGs. The positively correlated amino acid transport metabolism COGs are linked to saccharopine dehydrogenase NADP binding domain, component of the transport system for branched-chain amino acids and EamA like transporter family, while PFAM Lysine exporter protein (LYSE YGGA), PFAM Aminotransferase class I and II and Glutamate synthase domain 2 are negatively correlated to group 1. In addition to these genes, other sequences are correlated to the species of group 1 but with a lower statistical significance (Scoary, Benjamini-Hochberg's method corrected *p-value* < *0.05*; without Bonferroni's correction). Orthogroups related to *motA*, *tolQ, exbB, exbD* and *tonB* (TonB functions) are negatively related to group 1 and absent in all these species. However, another orthogroup encoding for MotA/TolQ/ExbB proton channel family results positively correlated with group 1 suggesting a specific function of different orthologues related to different *Arcobacter* species as already suggested for *A. butzleri* [16,78]. Moreover, another positively correlated orthogroup is linked to β-lactamase activity, present in all species of group 1 indicating antibiotic resistance-related genes [79]. The presence of antibiotic resistance traits in species considered of clinical and clinical-veterinary interest suggests a potential risk for human and animals health.

Overall, the results support an evolutionary adaptation of animals-related species through the loss of certain genes linked to basic metabolism and the maintenance of others.

## 4 Conclusions

The analysis performed in this study showed different genome sizes and genomic content in the species belonging to the *Arcobacteraceae* family, pointing towards as level of host adaptation or reservoir specificity. However, data show a large presence of cloud genes in their pangenome. Results obtained allowed the detection of genomic traits related to different groups of *Arcobacter*. Moreover, the comparative analysis of the genomes allowed to assess the pangenome partitions between groups, as well a smaller genome sizes of the animal-related species.

The genome partitions (core, cloud and soft genome) do not differ from other species belonging to other bacterial genera like *Campylobacter* spp., suggesting the existence of a single bacterial genus “*Arcobacter*”. This consideration is supported by comparative genomics analysis and ANI evaluation of species belonging to hypothesized genera (groups).

The moderate correlation of some PVGs to species related to animals and human clinical cases suggests the need to assess other gene candidates for the detection of pathogenic *Arcobacter* species. However, at present, evolutionary, and phylogenetic studies are still hampered by a wide presence of hypothetical proteins. Another limit is the absence in the databases of whole genomes and raw reads of multiple strains per species. Despite these limitations it was possible to highlight the smaller genome size of species considered animal and human clinical-related together with the detection of specific gene classes and the loss of others. This suggests an evolutionary specialization of a part of the species to animal hosts, leading to speculate about a link with the underestimation in clinical cases of *Arcobacter* spp. infections caused by the difficulty of *in vitro* cultivation. Moreover, the maintenance and presence of specific sequences demonstrate the importance of pathways for specific bacterial metabolism and lifestyle linked to different environmental niches. The study of 20 *Arcobacter* species type strains allowed the assessment of the *Arcobacteraceae* pangenome focused on species isolated from land animals and humans, with the detection of some specific sequences linked to specific ecological niches. The increase in the coming years of additional complete genome sequences available and of information relating to *Arcobacter* spp. strains and species will lead to new information favouring subsequent comparative studies at whole genome level.

## Author contribution statement

Davide Buzzanca: Conceived and designed the experiments; Performed the experiments; Analyzed and interpreted the data; Wrote the paper.

Pieter-Jan Kerkhof: Conceived and designed the experiments; Performed the experiments; Analyzed and interpreted the data.

Valentina Alessandria: Analyzed and interpreted the data; Wrote the paper.

Kalliopi Rantsiou: Analyzed and interpreted the data; Wrote the paper.

Kurt Houf: Conceived and designed the experiments; Analyzed and interpreted the data; Contributed reagents, materials, analysis tools or data; Wrote the paper.

## Data availability statement

Data associated with this study has been deposited at Part of the whole genomes raw sequence reads were collected from Sequence Read Archive (SRA) ENA (https://www.ebi.ac.uk/ena/browser/home), while the reads of the genomes sequenced in this work were uploaded to the NCBI bioproject PRJNA808439 (https://www.ncbi.nlm.nih.gov/). The accession numbers are shown in Table 1. The genomes assemblies were uploaded on Zenodo (https://zenodo.org/), to the DOI 10.5281/zenodo.6669654.

## Financing

Authors declare not to have financial support for this work, except for a ministerial PhD scholarship of Torino University for the first author.

## Declaration of competing interest

The authors declare that they have no known competing financial interests or personal relationships that could have appeared to influence the work reported in this paper.

## Abbreviations

Average Nucleotide Identity: ANI
Clusters of Orthologous Genes: COGs
Clustered Regularly Interspaced Short Palindromic Repeats: CRISPRs
Kyoto Encyclopedia of Genes and Genomes: KEGG
lipopolysaccharide: LPS
orthogroup: OG
putative virulence genes: PVGs
Single-copy Core Genes: SCGs
Sequence Read Archive: SRA
