# Supplementary material for: Arcobacteraceae comparative genome analysis demonstrates genome heterogeneity and reduction in species isolated from animals and associated with human illness
Source: Heliyon. 2023 Jun 27;9(7):e17652. doi: 10.1016/j.heliyon.2023.e17652 (PMC10336517; doi:10.1016/j.heliyon.2023.e17652)
Supplement: Multimedia component 1 [file mmc1.pdf]

Supplementary figures

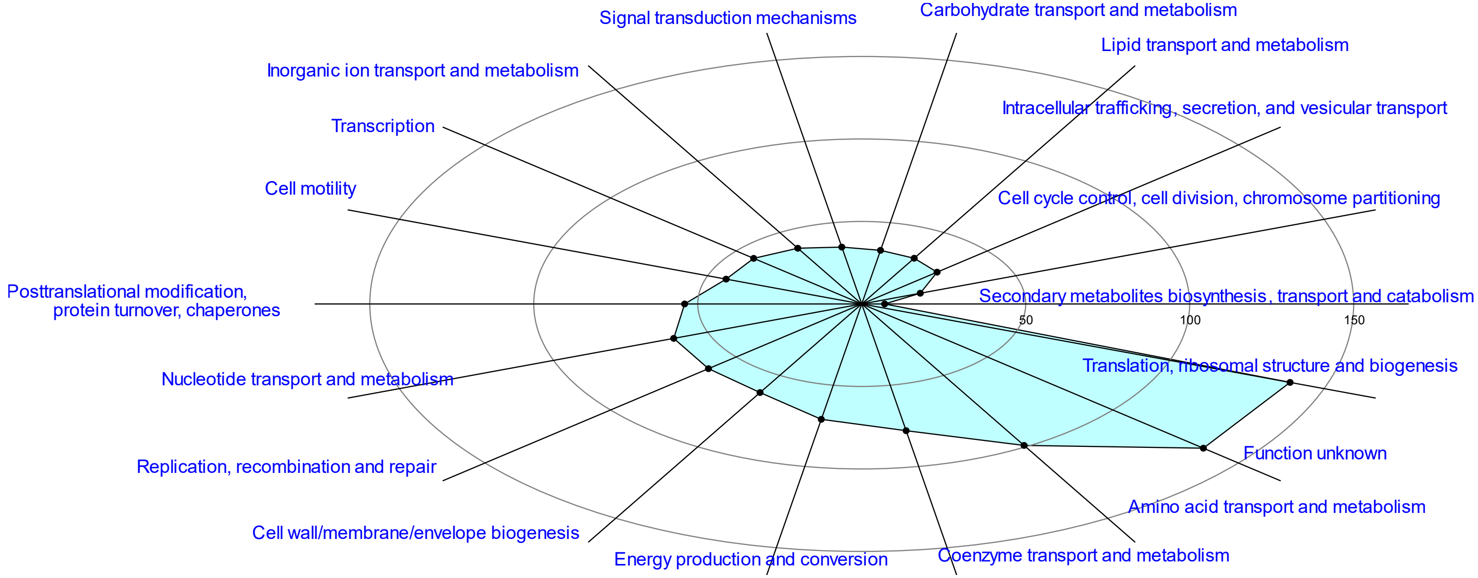

Supplementary figure 1. Orthogroups pathway present in all genomes. The figure shows the number of COGs codes relative to orthogroups present in all *Arcobacteraceae* species object of study.

%

64.00	66.00	70.00	73.00	75.00	77.00	80.00	82.00	85.00	87.00	90.00	95.00	100.00

< 74%

group 1
group 2
group 3
group 4
group 5
outgroup

	A. trophiarum LMG 25534T	*A. suis* LMG 26152T	*A. skirrowii* LMG 6621T	*A. venerupis* LMG 26156T	*A. ellisii* LMG 26155T	*A. porcinus* LMG 22487T	*A. butzleri* LMG 10828T	*A. nitrofigilis* LMG 7704T	*A. vandammei* LMG 31429T	*A. faecis* LMG28519T	*A. bivalviorum* LMG26154T	*A. halophilus* CCUG 53805T	*A. thereius* LMG24486T	*C. jejuni* NCTC11168	*A. cryaerophilus* LMG 10829	*A. lanthieri* LMG28516T	*A. vitoriensis* LMG30050T	*H. pylori* MT5135	*A. cibarius* LMG 21996T	*A. lacus* LMG 29062T	*A. molluscorum* LMG 25693T	*A. cryaerophilus* LMG 24291T	*A. mytili* LMG 24559T
A. trophiarum LMG 25534T	100.00																						
A. suis LMG 26152T	75.93	100.00																					
A. skirrowii LMG 6621T	80.76	76.56	100.00																				
A. venerupis LMG 26156T	75.77	86.76	76.15	100.00																			
A. ellisii LMG 26155T	76.78	83.16	77.82	82.43	100.00																		
A. porcinus LMG 22487T	79.92	76.38	81.35	75.87	77.19	100.00																	
A. butzleri LMG 10828T	77.63	80.82	78.54	80.15	82.79	77.65	100.00																
A. nitrofigilis LMG 7704T	73.23	75.51	73.72	76.09	75.24	73.75	75.28	100.00															
A. vandammei LMG 31429T	80.90	76.79	80.29	76.32	77.61	79.95	78.77	73.54	100.00														
A. faecis LMG28519T	81.21	77.01	80.52	76.71	77.85	79.67	79.27	74.04	85.02	100.00													
A. bivalviorum LMG26154T	73.50	75.85	74.23	75.46	76.04	74.46	75.49	75.04	74.09	74.26	100.00												
A. halophilus CCUG 53805T	73.57	75.53	74.14	75.30	75.83	74.52	75.47	74.77	74.27	74.05	76.39	100.00											
A. thereius LMG24486T	79.76	76.40	81.48	75.80	77.07	93.37	77.76	73.79	79.93	79.71	74.63	74.21	100.00										
C. jejuni NCTC11168	67.36	67.42	67.46	66.85	68.13	67.71	67.44	67.22	67.79	67.50	67.23	67.38	67.61	100.00									
A. cryaerophilus LMG 10829	85.38	76.85	84.15	76.30	77.95	80.50	78.93	73.57	81.40	81.89	74.03	74.44	80.42	67.37	100.00								
A. lanthieri LMG28516T	80.11	77.67	79.59	77.39	78.84	79.37	80.16	74.37	80.01	81.05	74.78	74.42	79.51	67.60	80.62	100.00							
A. vitoriensis LMG30050T	80.10	78.07	79.78	77.42	78.74	79.16	80.98	74.07	80.28	80.90	74.54	74.25	79.36	67.23	80.92	87.70	100.00						
H. pylori MT5135	64.89	64.63	66.30	64.90	65.25	65.22	66.12	65.21	64.84	66.25	64.68	65.27	65.18	65.37	64.57	65.78	65.27	100.00					
A. cibarius LMG 21996T	79.79	76.95	80.21	76.85	78.41	79.28	78.96	74.08	82.94	84.86	74.39	74.15	79.46	67.65	81.59	79.73	79.86	65.70	100.00				
A. lacus LMG 29062T	77.55	80.70	78.65	80.20	82.89	77.92	94.41	75.24	78.46	79.23	75.69	75.40	77.88	67.87	78.90	80.37	80.69	65.28	79.04	100.00			
A. molluscorum LMG 25693T	73.98	75.79	74.05	75.46	76.10	74.73	75.78	75.30	74.25	74.38	75.82	80.39	74.67	67.52	74.31	74.66	74.62	65.12	74.47	75.58	100.00		
A. cryaerophilus LMG 24291T	85.62	76.88	82.73	76.66	77.89	80.09	78.53	73.75	81.41	82.10	74.05	74.43	80.17	67.96	92.72	80.94	81.03	64.73	81.14	78.63	74.34	100.00	
A. mytili LMG 24559T	74.62	76.35	75.10	75.98	76.55	75.04	76.78	75.12	74.92	75.42	76.98	80.69	75.09	67.69	74.98	75.70	75.32	64.90	75.24	76.31	80.20	75.04	100.00

Supplementary figure 2. ANI values of the pair comparison of the different strains of *Arcobacter* spp. In the figure are indicated the species names and the correspondent group by different colors (1 to 5). Values below 74% are indicated by framed boxes. *C. jejuni* and *H. pylori* are included in the figure.

Gene Partitions	% Genomes	Roary	Panaroo	PPanGGOLin
Core	99 - 100	296	505	269
Soft core	95 - 99	43	90	326
Shell genes	15 - 95	1839	1965	366
Cloud genes	0 - 15	21237	13629	23178

A

B

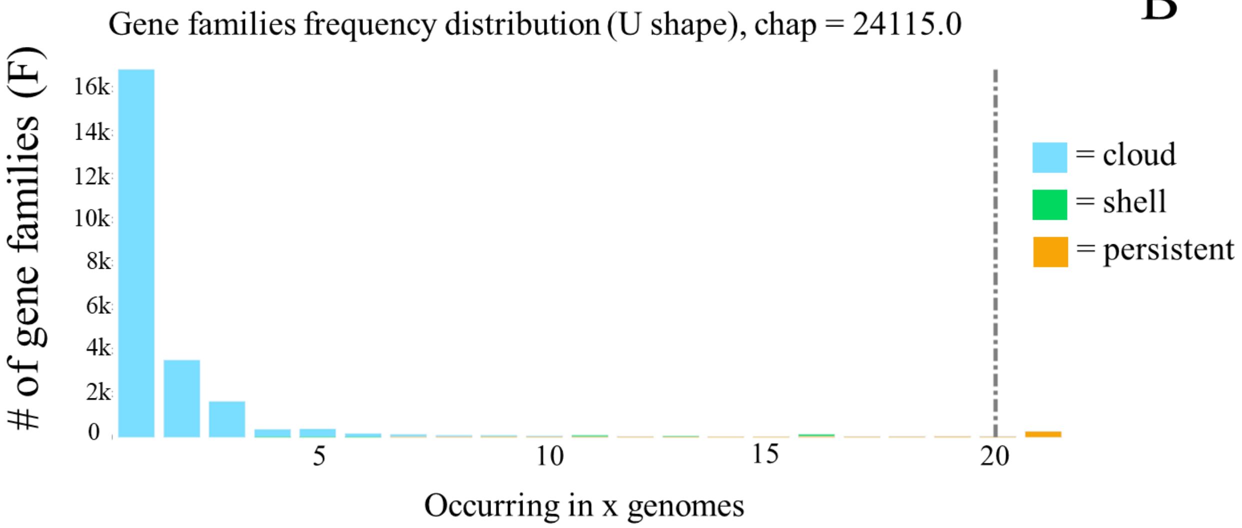

Supplementary figure 3. Information about genome partitions. The table **(A)** shows the different gene partitions (e.g. core genes) obtained with different tools. The histogram **(B)** shows gene families frequency. Shell and persistent genes are indicated respectively by green and orange bar on the right. The cloud genes are indicated by light blue bars.

Supplementary tables

Supplementary table 1. General information about *Arcobacteraceae* genomes. In the table are indicated different information about genomes general information (Quast), coverage (reads nr. * reads length)/genome size) and CRISPR/CAS sequences number. In the first line under the name of the strains the group to which they belong is indicated.

	A. bivalviorum LMG26154^T	*A. butzleri* LMG10828^T	*A. cibarius* LMG 21996^T	*A. cryaerophilus* LMG24291^T	*A. cryaerophilus* LMG10829	*A. ellisii* LMG26155^T	*A. faecis* LMG28519^T	*A. halophilus* CCUG53805^T
group	4	1	1	1	1	2	1	3
nr. contigs (>= 0 bp)	23	27	70	29	27	42	65	49
nr. contigs (>= 1000 bp)	22	23	60	23	23	37	56	41
nr. contigs (>= 5000 bp)	17	20	40	21	22	27	33	29
nr. contigs (>= 10000 bp)	15	18	34	19	19	23	30	23
nr. contigs (>= 25000 bp)	13	16	23	16	15	20	23	23
nr. contigs (>= 50000 bp)	12	11	10	14	12	17	16	17
Total length (>= 0 Mbp)	2.66	2.30	2.15	2.08	2.01	2.75	2.39	2.76
Total length (>= 1000 Mbp)	2.66	2.30	2.14	2.08	2.01	2.75	2.38	2.75
Total length (>= 5000 Mbp)	2.65	2.29	2.09	2.07	2.01	2.72	2.34	2.72
Total length (>= 10000 Mbp)	2.64	2.28	2.05	2.06	1.99	2.70	2.32	2.68
Total length (>= 25000 Mbp)	2.61	2.25	1.86	2.01	1.93	2.65	2.22	2.68
Total length (>= 50000 Mbp)	2.58	2.04	1.39	1.92	1.83	2.54	1.97	2.46
Total length Mbp	2.66	2.30	2.15	2.08	2.01	2.75	2.39	2.76
coverage	353	217	197	441	471	277	348	333
N50	247420	234980	122275	194390	165914	177151	123980	177704
N75	178940	112969	35326	101744	104157	110030	75048	88435
L50	4	4	6	5	4	6	7	6
L75	7	8	16	9	8	11	14	12
GC (%)	28	26.87	26.78	27.19	27.39	26.76	26.96	27.43
CRISPR	0	0	2	2	1	5	3	5
CAS	2	0	4	0	1	1	3	4
CRISPR/CAS associated sequences	0	0	1	0	0	0	1	1

	A. lacus LMG29062T	*A. lanthieri* LMG28516T	*A. molluscorum* LMG25693T	*A. mytili* LMG24559T	*A. nitrofigilis* LMG 7704T	*A. porcinus* LMG22487T	*A. skirrowii* LMG6621T	*A. suis* LMG26152
group	1	1	3	3	5	1	1	2

nr. contigs (>= 0 bp)	23	30	68	59	1	29	19	57
nr. contigs (>= 1000 bp)	20	25	54	50	1	28	18	52
nr. contigs (>= 5000 bp)	18	18	36	42	1	24	14	36
nr. contigs (>= 10000 bp)	17	17	34	35	1	19	11	30
nr. contigs (>= 25000 bp)	15	14	31	29	1	15	9	22
nr. contigs (>= 50000 bp)	14	11	22	17	1	10	8	17
Total length (>= 0 Mbp)	2.22	2.24	2.73	2.97	3.19	1.79	1.96	2.58
Total length (>= 1000 Mbp)	2.22	2.23	2.72	2.97	3.19	1.79	1.96	2.58
Total length (>= 5000 Mbp)	2.21	2.22	2.67	2.95	3.19	1.79	1.95	2.54
Total length (>= 10000 Mbp)	2.21	2.21	2.66	2.90	3.19	1.75	1.93	2.51
Total length (>= 25000 Mbp)	2.18	2.17	2.61	2.81	3.19	1.68	1.89	2.35
Total length (>= 50000 Mbp)	2.13	2.04	2.28	2.41	3.19	1.49	1.84	2.19
Total length Mbp	2.22	2.24	2.73	2.97	3.19	1.79	1.96	2.58
coverage	75	365	464	382	Ref. genome	74	362	342
N50	207786	369058	88441	164261	3192235	140725	305996	142035
N75	107947	91794	60477	77475	3192235	75594	205942	75047
L50	5	2	9	7	1	4	2	14
L75	8	7	18	15	1	8	4	7
GC (%)	26.78	26.41	26.03	26.35	28.36	27.17	27.66	27.23
CRISPR	0	2	4	1	1	1	2	1
CAS	0	3	4	7	1	1	1	6
CRISPR/CAS associated sequences	0	1	2	1	1	0	1	1

	A. thereius LMG24486^T	*A. trophiarum* LMG25534^T	*A. vandammei* LMG31429^T	*A. venerupis* LMG26156^T	*A. vitoriensis* LMG30050^T	*C. jejuni* NCTC11168	*H. pylori* MT5135
group	1	1	1	2	1	-	-
nr. contigs (>= 0 bp)	9	35	56	89	40	1	1
nr. contigs (>= 1000 bp)	8	31	45	72	36	1	1
nr. contigs (>= 5000 bp)	7	27	34	57	32	1	1
nr. contigs (>= 10000 bp)	5	25	29	52	29	1	1
nr. contigs (>= 25000 bp)	5	19	21	38	24	1	1
nr. contigs (>= 50000 bp)	4	13	15	26	18	1	1

Total length (>= 0 Mbp)	1.90	1.87	2.21	3.16	2.42	1.64	1.62
Total length (>= 1000 Mbp)	1.90	1.87	2.20	3.15	2.41	1.64	1.62
Total length (>= 5000 Mbp)	1.90	1.86	2.17	3.12	2.41	1.64	1.62
Total length (>= 10000 Mbp)	1.89	1.85	2.13	3.08	2.39	1.64	1.62
Total length (>= 25000 Mbp)	1.89	1.74	1.99	2.84	2.31	1.64	1.62
Total length (>= 50000 Mbp)	1.85	1.54	1.76	2.42	2.11	1.64	1.62
Total length Mbp	1.90	1.87	2.21	3.16	2.42	1.64	1.62
GC (%)	26.92	28.04	27.6	27.17	27.02	30.55	39.28
coverage	59	253	215	149	339	Ref. genome	Ref. genome
N50	509145	122603	107602	82335	127074	1641481	1615199
N75	492236	63280	68457	53970	81644	1641481	1615199
L50	2	5	8	14	8	1	1
L75	3	11	14	26	14	1	1
CRISPR	5	0	2	0	1	2	1
CAS	2	0	3	5	3	1	0
CRISPR/CAS associated sequences	1	0	1	0	0	1	0

Supplementary table 2. Clusters of Orthologous Genes functions number. The table shows the number of annotated orthogroups COGs obtained from EggNOG mapper analysis. At the end of the table are indicated the coding of the letters relating to the different classes.

	A	C	D	E	F	G	H	I	J	K	L	M	N	O	P	Q	S	T	U	V	Z
A. bivalviorum	1	193	25	204	85	78	135	55	158	122	99	145	80	101	149	36	368	248	59	32	0
A. butzleri	1	154	26	159	76	73	113	45	152	118	97	153	84	87	136	27	363	173	54	33	1
A. cibarius	0	137	25	134	73	60	114	45	155	80	155	140	73	88	110	20	309	158	62	26	0
A. cryaerophilus (T)	0	133	26	134	73	65	119	55	152	78	109	138	76	83	106	30	318	128	57	27	0
A. cryaerophilus	0	130	28	139	72	62	115	52	154	74	97	139	78	82	108	30	314	133	47	24	0
A. ellisii	1	208	29	168	81	70	124	57	159	128	140	156	84	113	142	40	424	231	57	34	1
A. faecis	0	155	26	145	75	63	120	53	153	113	124	157	90	83	140	29	357	188	51	28	0
A. halophilus	1	225	25	189	83	78	141	57	157	115	107	132	88	88	171	33	381	250	57	33	0
A. lacus	1	138	22	166	75	72	102	69	151	118	104	131	71	89	130	43	332	150	46	34	1
A. lanthieri	1	137	24	154	71	68	116	50	150	103	118	140	87	85	168	31	320	151	56	25	0
A. molluscorum	1	235	19	199	79	75	143	52	158	126	114	145	79	100	141	31	413	217	61	34	0
A. mytili	1	223	24	199	80	77	143	66	154	121	111	160	84	94	167	46	372	247	49	35	0
A. nitrofigilis	0	265	23	256	92	128	153	72	163	164	125	186	88	112	208	60	496	251	75	38	0
A. porcinus	0	122	23	134	69	54	112	43	153	73	98	113	62	79	96	16	268	93	49	17	0
A. skirrowii	0	126	24	136	70	59	117	40	149	74	109	120	69	85	114	20	315	105	48	18	0
A. suis	1	210	23	164	78	72	130	64	161	122	102	148	81	97	133	40	388	202	55	32	1
A. thereius	0	120	25	133	70	55	115	38	153	82	114	110	65	80	107	18	271	95	44	24	0
A. trophiarum	0	136	23	132	71	61	116	50	149	73	101	124	68	80	102	25	277	105	55	23	0
A. vandammei	0	135	27	132	71	62	117	52	155	90	138	133	62	76	108	24	334	124	53	41	0
A. venerupis	1	253	28	233	87	102	143	66	167	153	111	183	103	109	184	50	464	254	79	52	1
A. vitoriensis	1	140	27	164	74	74	119	53	151	130	100	153	75	86	195	32	340	152	55	32	0

A
RNA processing and modification
C
Energy production and conversion
D
Cell cycle control, cell division, chromosome partitioning
E

J
Translation, ribosomal structure and biogenesis
K
Transcription
L
Replication, recombination and repair
M

Amino acid transport and metabolism	Cell wall/membrane/envelope biogenesis
F	N
Nucleotide transport and metabolism	Cell motility
G	O
Carbohydrate transport and metabolism	Posttranslational modification, protein turnover, chaperones
H	P
Coenzyme transport and metabolism	Inorganic ion transport and metabolism
I	Q
Lipid transport and metabolism	Secondary metabolites biosynthesis, transport and catabolism
S	V
Function unknown	Defense mechanisms
T	Z
Signal transduction mechanisms	Cytoskeleton
U	
Intracellular trafficking, secretion, and vesicular transport	

Supplementary table 3. Presence/absence of secondary metabolites related genes. The table shows presence (1) and absence (0) of secondary metabolites related sequences in the 21 genomes object of study.

	ectoine	RiPP-like	arylpolyene - resorcinol	ranthipeptide	thiopeptide	resorcinol	Acyl aminoacids	NRPS-T1PKS	redox cofactor	arylpolyene
A. bivalviorum LMG26154^T	1	1	1	0	0	0	0	0	0	0
A. ellisii LMG26155^T	1	0	0	1	0	0	0	0	0	0
A. halophilus CCUG53805^T	1	1	0	0	0	0	0	0	0	0
A. lathieri LMG28516^T	0	0	0	0	0	0	0	0	0	0
A. molluscorum LMG25693^T	1	0	0	1	0	0	0	0	0	0
A. mytili LMG24559^T	1	0	1	0	0	0	0	0	0	0
A. nitrofigilis LMG7704^T	1	0	1	0	0	0	0	1	1	0
A. suis LMG26152^T	0	0	0	1	0	0	0	0	0	0
A. trophiarum LMG25534^T	0	0	1	0	0	0	0	0	0	0
A. vandammei LMG31429^T	0	0	0	0	0	0	0	0	0	0
A. venerupis LMG26156^T	1	0	0	1	0	0	0	0	1	0
A. vitoriensis LMG30050^T	1	0	0	0	0	0	0	0	0	1
A. butzleri LMG10828^T	0	0	0	1	1	0	0	0	0	0
A. cibarius LMG21996^T	0	0	0	0	1	0	0	0	0	0

A. cryaerophilus LMG24291^{T}	1	0	1	0	1	1	1	0	0	0
A. cryaerophilus LMG10829	0	0	1	0	1	0	0	0	0	0
A. faecis LMG28519^{T}	0	0	0	0	1	0	1	0	0	0
A. lacus LMG29062^{T}	0	0	1	1	1	0	0	0	0	0
A. porcinus LMG24487^{T}	1	0	0	0	1	0	0	0	0	0
A. skirrowii LMG6621^{T}	1	0	0	0	1	0	0	0	0	0
A. thereius LMG24486^{T}	1	0	0	0	1	0	0	0	0	0
